# Differences in seasonal dynamics and pyrethroid resistance development among *Anopheles* Hyrcanus group species

**DOI:** 10.1186/s13071-024-06462-8

**Published:** 2024-10-05

**Authors:** Do Eun Lee, Jeong Heum Han, Gang Chan Lee, Junhyeong Choi, Wonyong Kwun, Si Hyeock Lee, Ju Hyeon Kim

**Affiliations:** 1 Present Address: Department of Agricultural Biotechnology, Seoul National University, Seoul, 08826 Republic of Korea; 2 Department of Tropical Medicine and Parasitology, Seoul National University College of Medicine, Seoul, 03080 Republic of Korea; 3 Research Institute of Agriculture and Life Sciences, Seoul National University, Seoul, 08826 Republic of Korea; 4 Institute of Endemic Diseases, Seoul National University College of Medicine, Seoul, 03080 Republic of Korea

**Keywords:** *Anopheles* Hyrcanus group, Species composition, Seasonal dynamics, Pyrethroid resistance, λ-cyhalothrin, *Kdr* mutation, Baseline susceptibility, Voltage-sensitive sodium channel

## Abstract

**Background:**

The *Anopheles* Hyrcanus group, which transmits *Plasmodium vivax*, consists of six confirmed species in South Korea. An epidemiological study revealed differences in the seasonal occurrence patterns of each species. Pyrethroid resistance in *An. sinensis* dates back to the early 2000s, whereas information on pyrethroid resistance in other species is lacking despite their greater significance in malaria epidemiology.

**Methods:**

*Anopheles* mosquitoes were collected from two malaria-endemic regions in South Korea for 2 years and their knockdown resistance (*kdr*) mutations were genotyped. The larval susceptibility to λ-cyhalothrin was compared in six *Anopheles* species and its seasonal changes in three species were investigated. The full-length sequences of the voltage-sensitive sodium channel (VSSC) were compared across six species to evaluate potential target-site insensitivity. The contribution of the *kdr* mutation to phenotypic resistance was confirmed by comparing median lethal time (LT_50_) to λ-cyhalothrin between populations of *Anopheles belenrae* with distinct genotypes.

**Results:**

The composition and seasonal occurrence of rare species (*Anopheles kleini*, *Anopheles lestri*, and *Anopheles sineroides*) varied considerably, whereas *An. sinensis* occurs continuously throughout the season. A *kdr* mutation in the form of heterozygous allele was newly identified in *An. belenrae*, *An. lesteri*, *An. pullus*, and *An. sineroides*. The baseline susceptibility to λ-cyhalothrin was the highest in *An. belenrae*, followed by *An. lesteri*, *An. sineroides*, *An. kleini*, *An. pullus*, and *An. sinensis,* with median lethal concentration (LC_50_) values ranging from 6.0- to 73.5-fold higher than that of *An. belenrae.* The susceptibility of *An. sinensis* and *An. pullus* varied by season, whereas that of *An. belenrae* remained stable. The *kdr*-heterozygous *An. belenare* population exhibited 5.1 times higher LT_50_ than that of the susceptible population. Species-specific VSSC sequence differences were observed among the six species.

**Conclusions:**

Our findings suggest that the status and extent of pyrethroid resistance vary among *Anopheles* Hyrcanus group species. While *An. sinensis*, the predominant species, developed a considerable level of pyrethroid resistance through *kdr* mutation, the resistance levels of other species appeared to be less pronounced. Large-scale monitoring is crucial to fully understand species-specific seasonal occurrence and resistance status for effective management strategies, considering the ongoing impact of climate change on their vectorial capacity.

**Graphical Abstract:**

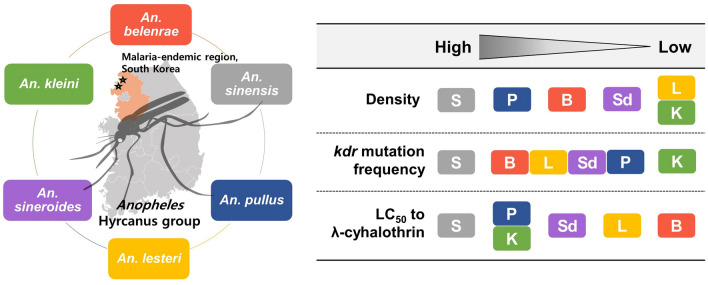

**Supplementary Information:**

The online version contains supplementary material available at 10.1186/s13071-024-06462-8.

## Background

The *Anopheles* Hyrcanus group, a vector of *Plasmodium vivax* in East Asia, comprises six species (*Anopheles belenrae*, *Anopheles kleini*, *Anopheles lesteri*, *Anopheles pullus*, *Anopheles sinensis*, and *Anopheles sineroides*). All these species are present in the northern Gyeonggi province of South Korea [1, 2]. Epidemiological studies in northern Gyeonggi province have revealed that *An. pullus* was prevalent in early summer, *An. kleini* in mid-summer, and *An. sinensis* from mid-summer to late summer [1]. Comparative studies of the vector competence of *An*. *sinensis*, *An. kleini*, *An. pullus,* and *An. belenrae* indicated that *An. kleini* had the strongest vector competence, whereas *An. sinensis* exhibited the lowest susceptibility to *P. vivax* [3, 4]. Therefore, obtaining information on the seasonal abundance of *Anopheles* Hyrcanus group member species in malaria-endemic areas is crucial for understanding the population dynamics of vector populations and the epidemiology of malaria.

Pyrethroid resistance in the field population of *An. sinensis*, the predominant species, has been consistently reported in South Korea since 2001 [5]. Resistance to permethrin and deltamethrin in *An. sinensis* increased significantly over time, with resistance ratios of 15.6 and 22.6, respectively [6]. Pyrethroid resistance mutations were detected in South Korea before the early 2000s, with frequencies ranging from 25% to 97% [7]. Despite their distinct physiological and ecological characteristics related to vector competence, information on the differences in insecticide resistance traits among minor species of the *Anopheles* Hyrcanus group is scarce. This scarcity is primarily due to the challenges in distinguishing individual species from overlapping habitats. No susceptibility tests have been conducted on *Anopheles* species other than *An. sinensis*. However, they have a lower population density than *An. sinensis*, and assessing the pyrethroid resistance status of other species with higher vector competence is crucial for establishing an effective management strategy and preparing for potential outbreaks caused by climate change.

The development of pyrethroid resistance in mosquitoes primarily occurs through two major mechanisms: target alterations and metabolic detoxifications [8]. One of the most common target site alterations observed in *Anopheles* mosquitoes involves mutations in the voltage-sensitive sodium channel (*vssc*) gene, collectively known as knockdown resistance (*kdr*) mutations (L1014F/C/S and N1013S, N1575Y, and V1010L mutations) [9]. The L1014F mutation has been extensively reported in *An. sinensis*. Its absence in the other five species was documented in a single genotyping study a decade ago [10]. In addition to the major *kdr* mutation (L1014F) found in most insects, additional mutations such as M918T in *Musca domestica* and V1016I and F1534C in *Aedes aegypti* have been reported to be potentially associated with resistance [11]. Therefore, more comprehensive mutation detection in current mosquito populations is necessary to assess the recent pyrethroid resistance status across individual species in the *Anopheles* Hyrcanus group.

To address this gap in our understanding, the present study aimed to monitor the seasonal occurrence of *Anopheles* species in malaria-endemic Paju and Gimpo areas over 2 years using conventional species identification methods and to determine their *kdr* mutation frequencies. Additionally, we compared the larval susceptibilities of the six species in the *Anopheles* Hyrcanus group with λ-cyhalothrin through bioassays. Finally, we conducted an extensive search for VSSC mutations potentially associated with pyrethroid resistance across six species.

## Methods

### Mosquito collection and species identification

Blood-fed *Anopheles* mosquitoes were collected from Gyeonggi province, South Korea (Paju-1: 37.871385, 126.772513; Paju-2: 37.819755, 126.694960; and Gimpo: 37.708802, 126.643029), from May 2022 to September 2023 using black light traps (BT Global, South Korea) with an attractant overnight in a cowshed. Blood-fed mosquitoes were moved into 30 cm^3^ cages and provided with 10% sucrose for one day. Oviposition was induced by isolating individual *Anopheles* mosquitoes using a single wing cut in a paper cup containing 50 ml of water. Molecular identification was performed using DNA released from each wing by 10 µl of DNA-releasing buffer (5% of DMSO, 5% of PEG 200, 20 mM of NaOH, and 1 mM of EDTA). A multiplex polymerase chain reaction (PCR) method [12] with modifications to four primer sequences (see Additional file 1: Tables S.1) was used to identify six *Anopheles* Hyrcanus group species included in this study. To examine the seasonal distribution of these species, all individual mosquitoes collected at each site were identified using the multiplex PCR method during weeks 21–37 (Paju 2022), weeks 28–39 (Paju 2023), weeks 21–40 (Gimpo 2022), and weeks 28–40 (Gimpo 2023).

### Genotyping of *kdr* mutation in six *Anopheles* Hyrcanus group species

Randomly selected mosquitoes collected from the Paju area in 2022 and 2023 were individually genotyped using the primer sets from a previous study (An_vssc-F: GACTTCATGCACTCCTTCATG; An_vssc-R: GTTGGTGCAGAGAGCGATGA) [13]. The 20 µl reaction mixture composed of 0.8 µl of released DNA template, 0.5 mM of each primer, 0.2 mM of dNTP, 2 µl of 10X buffer, and 0.1 µl of EX taq polymerase (Takara, Seoul, Korea). After 35 cycles of 95 ℃ for 30 s, 55 ℃ for 30 s, and 72 ℃ for 50 s, amplicons were purified and then sequenced using ABI3730xl. For mutation frequency calculation, individuals with homozygous susceptible and resistant alleles were recorded as 0 and 1, respectively, whereas heterozygous individuals were recorded as 0.5.

### λ-cyhalothrin susceptibility of six *Anopheles* species larvae

Λ-cyhalothrin is a type II pyrethroid insecticide that has been widely used for mosquito control in South Korea. It was selected in this study to estimate pyrethroid resistance level in *Anopheles* larvae, as previous research found it to be the most effective against *An. sinensis* larvae among eight different pyrethroids tested [6]. Mosquito larvae hatched from eggs were reared under controlled conditions at 25 ℃, 14 h light and 10 h dark cycle (14L:10D), and 60% humidity. The filter paper was placed in the breeding dish, and tetramin flakes were provided daily to the F_1_ generation larvae. λ-cyhalothrin susceptibility tests were conducted on F_1_ larvae of mosquitoes collected from the Paju area from August to September, where all six species were confirmed to be present. Larval bioassays were conducted with 10–15 third instar larvae following World Health Organization (WHO) guidelines [14]. Four concentrations (0, 10, 100, and 1000 ppb) of λ-cyhalothrin were treated for 24 h and the mortality was observed. Mosquito species (*An. sinensis* and *An. pullus*), showing less than 50% mortality at 1000 ppb, were additionally treated with a 10,000 ppb concentration. To prepare these working solutions, 0.5 ml of the serially diluted 1–1000 ppm stock solutions of λ-cyhalothrin (analytical standard, Sigma-Aldrich 31058) in acetone were diluted 100-fold with 49.5 ml of distilled water using a 5-oz. paper cup. Mortality was observed after 24 h and the larvae were counted as “dead” if they could not escape from the forceps when held. To evaluate seasonal changes in susceptibility, bioassays will be performed on the collection groups at weeks 23, 24, 35, and 36 in 2022.

### Cloning of *vssc* gene

The full-length *vssc* gene, which encodes the target of pyrethroid insecticides, was amplified and cloned to identify point mutations or transcript variants potentially causing insensitivity to pyrethroids across the six *Anopheles* species. A 6.4 kb-sized fragment (using primer An_vE1-0F and An_vE32-6399R in Table 1) was amplified from the cDNA of each species using Advantage 2 Taq polymerase (Takara, Seoul, Korea) in a 10 µl reaction for 30 cycles. Subsequently, nested PCRs were conducted to obtain four overlapping cDNA fragments (1.5–1.8 kb) covering the entire sequences using four different primer sets (Table 1). Amplified fragments were ligated into the pGEM-T easy vector (Promega, WI, USA) using T4 DNA ligase (Promega) and transformed into DH5a competent cells at 42 ℃ for 45 s. Plasmids were extracted from overnight cultured colonies using a mini prep kit (Bioneer, Daejeon, South Korea) and sequenced. The sequences were aligned using CLC Main Workbench 8.1.2 (QIAGEN) with the reference sequence of *An. sinensis* (ASIS024373) obtained from VectorBase.

**Table 1:** Primer sets used for cloning the *vssc* gene

Primer name	F/R	Sequence (5′-3′)	Position (nt)
An_vE1-0F	F	ATGACCGAAGACTCCGATTCG	0
An_vE1-19F	F	CGATATCTGAGGAAGAACGTAG	19
An_vE13-1781R	R	GGCAGGTGTTCTTGTGCATC	1781
An_vE13-1702F	F	GGACGTTTCGTTGGTGTACC	1702
An_vE22-3290R	R	AGCTCATTTTCACCATGCTCTG	3290
An_vE22-3266F	F	CTGCAGAGCATGGTGAAAATG	3266
An_vE29-4886R	R	GCATRTTAAATCCGATGAACAACAT	4886
An_vE29-4712F	F	GTTCATGACAGAAGATCAGAAGA	4712
An_vE32-6377R	R	ATACTGGGCGATCGTGAGTG	6377
An_vE32-6399R	R	GACATCTGCCGTGCGTGATGT	6399

### Copy number validation of *vssc* gene

Given that duplication of the VSSC target protein may affect pyrethroid sensitivity, the copy number of *vssc* loci was assessed by quantitative real-time PCR (qPCR) using the LightCycler 96 (Roche, Basel, Switzerland). Single-copy genes such as ribosomal protein S7 and ribosomal protein S3 were used as references. gDNA was extracted separately from four to seven individuals of each of the six species. The reaction mixture (10 µl) contained 10 ng of gDNA template, 0.5 µM of each primer, and 5 µl of TB Green™ Premix Ex Taq™ II (Takara Biotechnology, Japan). qPCR for each species was conducted with technical replicates using the amplification condition of 95 °C denaturation for 1 min followed by 35 cycles of 95 °C for 15 s, 57 °C for 20 s, and 72 °C for 30 s.

### Median lethal time (LT_50_) of susceptible and heterozygous resistant *Anopheles belenrae*

To demonstrate that the *kdr* trait (L1014F mutation) confers pyrethroid resistance to *An. belenrae*, fourth instar larvae produced from females with the heterozygous *kdr* genotype were treated with 1 ppm λ-cyhalothrin. Mortality to λ-cyhalothrin was observed at 15, 20, 40, 60, 120, and 180 min after treatment and dead larvae were immediately frozen to release DNA individually. After mortality evaluation, the *kdr* genotype of all tested individual mosquitoes was investigated using the method described above (Mosquito collection and species identification). Individual larvae were sorted on the basis of their genotype and mortality responses. Mortality time curves were then generated for the larvae of each genotype, distinguishing between the homozygous susceptible and heterozygous groups. Finally, LT_50_ values were predicted and compared between the two groups.

### Data analysis

The median lethal concentration (LC_50_) of six *Anopheles* species and the LT_50_ of *An. belenrae* was calculated using logit analysis and a log-likelihood test was performed using POLO ver 2.0 (LeOra Software, Parma, Italy). To determine the statistical significance of seasonal changes in susceptibility among the three *Anopheles* species, an unpaired *t*-test was conducted using the Sidak-Bonferroni method. The data were visualized using SigmaPlot 10.0 (Systat Software Inc., CA, USA) and GraphPad Prism 6.0 (GraphPad Software, CA, USA). Sequence alignment was conducted using the CLC Main Workbench 8 (Qiagen, MD, USA).

## Results

### Seasonal distribution of *Anopheles* Hyrcanus group species

**Fig. 1:**
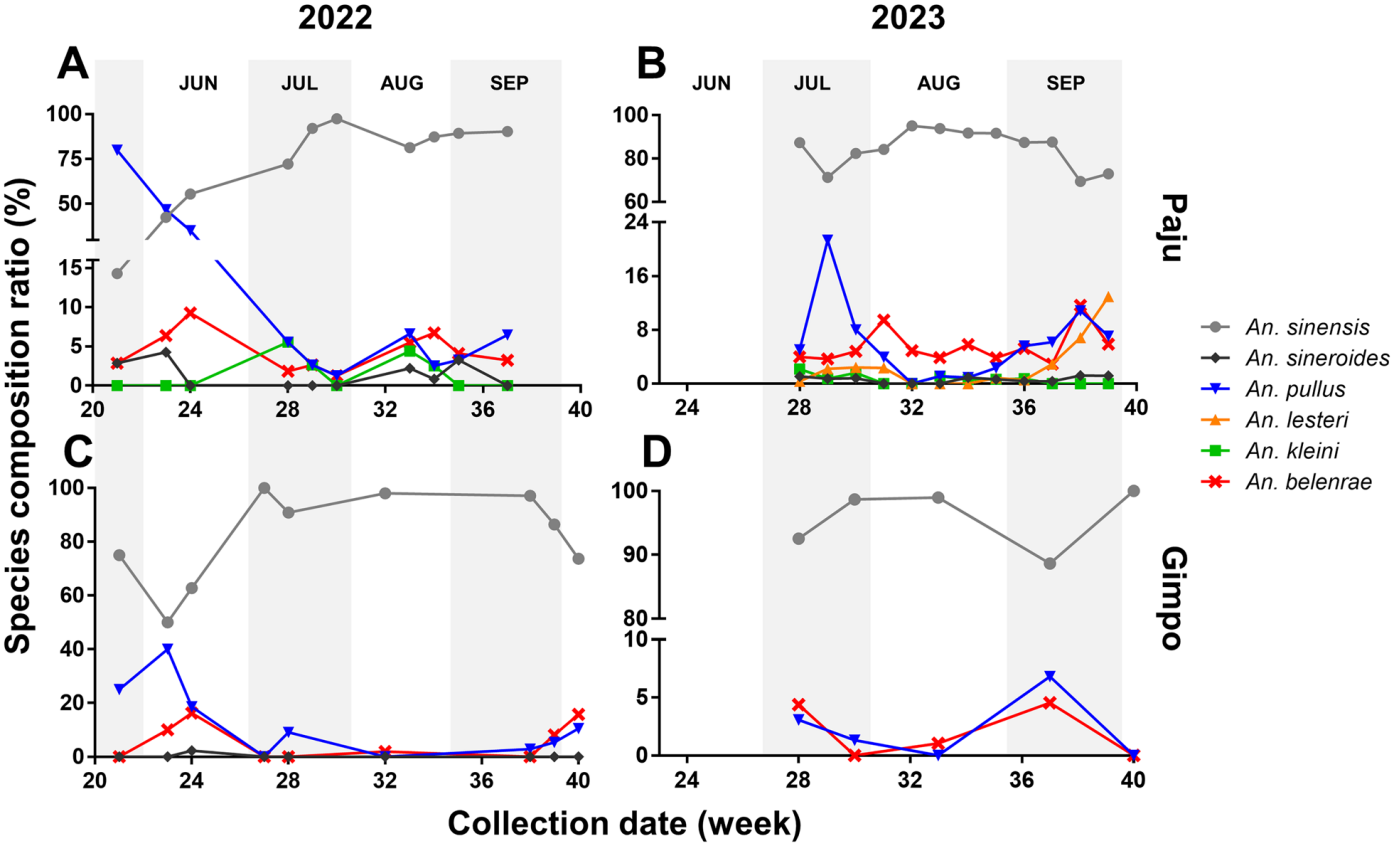
Species composition ratio of the *Anopheles* Hyrcanus group collected from Paju (**A** and **B**) and Gimpo (**C** and **D**) areas during 2022–2023

A total of five species were collected in 2022 from the Paju-1 site in Paju, excluding *An. lesteri*. *Anopheles sinensis*, *An. pullus*, and *An. belenrae* were consistently observed throughout the season (Fig. 1). *Anopheles pullus* dominated until June 4, after which *An. sinensis* became predominant, accounting for over 80% of the population from July, maintaining this trend until September 15. *Anopheles pullus* was the second most prevalent species, ranging from 1.3% (on July 26) to 80% (on May 25). *Anopheles belenrae* was observed at very low densities, ranging from 1.3% (on July 26) to 9.3% (on June 11). Moreover, *An. sineroides* was observed in both the early and late seasons, peaking at 4.3% (on June 4), whereas *An. kleini* was observed only from July to August, peaking at 5.6% (on July 10).

In 2023, collections were conducted at both Paju-1 and Paju-2, approximately 8 km apart. Similar to 2022, *An. sinensis* was the most abundant species, accounting for more than 50% of the total population from July to September, followed by *An. pullus,* peaking at 21.3% (on July 17). Unlike in 2022, *An. lesteri* was observed, with the simultaneous presence of all six species confirmed in July. The composition of *An. lesteri* reached 12.9% during the late season.

At Gimpo, collections were conducted in 2022 and 2023. Four species were collected during the 2022 collection, with *An. lesteri* and *An. sineroides* not observed. *Anopheles sinensis* was the only species observed throughout the season. The composition ratios of *An. pullus* and *An. belenrae* were the highest in the early season (40% and 16.3%, respectively), decreased in mid-summer, and then increased again in the late season (10.5% and 15.8%, respectively). In 2023, only three species (*An. sinensis*, *An. pullus*, and *An. belenrae*) were observed at Gimpo since the collection began in July.

### Frequency of *kdr* mutation (L1014F/C) in six *Anopheles* Hyrcanus group species

**Fig. 2:**
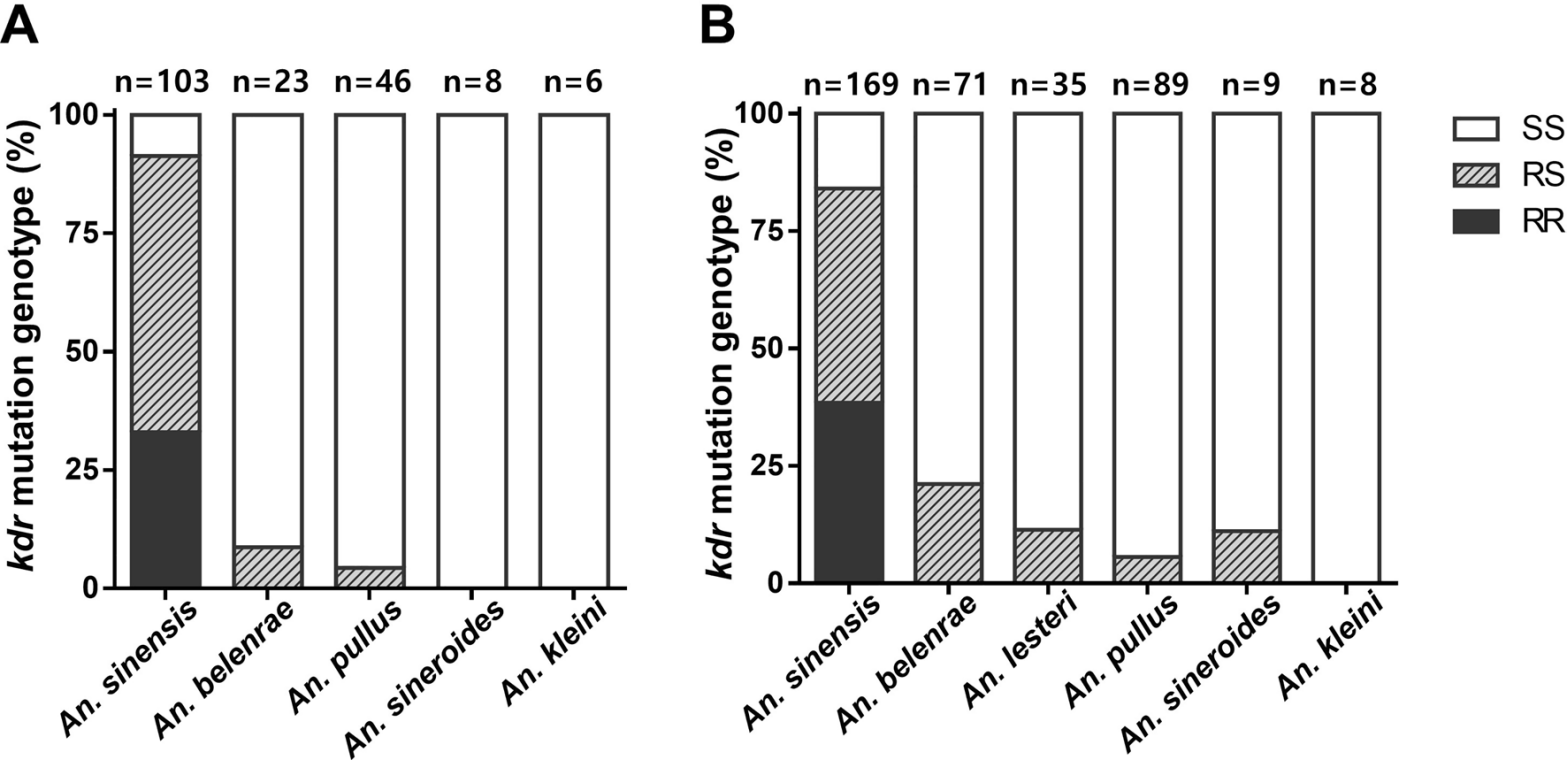
Percentage of mosquitoes with homozygous susceptible (SS), heterozygous (RS), and homozygous resistant (RR) alleles. Mosquitoes were collected from Paju in 2022 (**A**) and 2023 (**B**)

The *kdr* mutation allele was genotyped in 186 individuals from 5 species collected in 2022 (Table 2 and Fig. 2A) and 381 individuals from all 6 *Anopheles* species collected in 2023 in Paju (Table 3 and Fig. 2B). *Anopheles sinensis* was the only species exhibiting a homozygous resistance allele (FF/CF/CC) on all collection dates, which is consistent with previous findings [10]. Notably, the L1014F mutation was identified in *An. belenrae* and *An. pullus* for the first time, although at relatively low frequencies (8.7% and 4.3%, respectively) in the heterozygous form, compared to *An. sinensis* (58.2%) by 2022. Furthermore, the L1014F mutation was newly identified in *An*. *lesteri* and *An*. *sineroides* by 2023 and the L1014C mutation was detected in *An. belenrae* and *An. lesteri*. The frequency of the heterozygous genotype was the highest in *An. sinensis* (58.2% in 2022 and 45.6% in 2023), followed by *An. belenrae* (8.7% in 2022 and 21.2% in 2023) (Tables 2 and 3). Despite the larger population size of *An. pullus* compared with *An. belenrae* and *An. lesteri,* it showed relatively low frequencies of *kdr* mutation. Interestingly, *An. kleini* exhibited an absence of *kdr* mutations, which could be attributed to either the limited sample size examined or lower prevalence within the population.

**Table 2:** Number of mosquitoes with L1014F/C mutation in six *Anopheles* Hyrcanus group species collected from Paju at eight different timepoints in 2022

	1014 allele	04-JUN	11-JUN	10-JUL	26-JUL	18-AUG	26-AUG	01-SEP	15-SEP	Total
*An. blelnrae*	LL	3	5		1	5	4	2	1	21 (91.3%)
	LF			1			1			2 (8.7%)
*An. kleini*	LL	–	–	2	–	4	–	–	–	6 (100%)
*An. pullus*	LL	14	16	3	1	3		5	2	44 (95.7%)
	LF		2							2 (4.3%)
*An. sinensis*	LL	1	2	0	1	1	1	2	1	9 (8.7%)
	LF	8	6	3	6	8	1	9	3	44 (42.7%)
	LC	0	2	1	2	1	1	5	4	16 (15.5%)
	CF/FF	2	7	4	0	8	7	4	2	34 (33.0%)
*An. sineroides*	LL	2				2	1	3		8 (100%)

**Table 3:** Number of mosquitoes with L1014F/C mutation in six *Anopheles* Hyrcanus group species collected from Paju at six different timepoints in 2023

	1014 allele	24-JUL	16-AUG	27-AUG	03-SEP	13-SEP	20-SEP	Total
*An. belenrae*	LL	–	3	2	20	6	25	56 (78.9%)
	LF	–	0	2	4	1	2	9 (12.7%)
	LC	–	1	4	1	0	0	6 (8.5%)
*An. kleini*	LL	2	2	2	2	–	–	8 (100%)
*An. lesteri*	LL	4	–	1	2	8	16	31 (88.6%)
	LF	0	–	2	0	0	0	2 (5.7%)
	LC	0	–	1	0	0	1	2 (5.7%)
*An. pullus*	LL	12	1	9	25	16	21	84 (94.4%)
	LF	0	0	2	2	1	0	5 (5.6%)
*An. sinensis*	LL	3	1	2	9	8	4	27 (16.0%)
	LF	10	0	6	11	15	10	52 (30.8%)
	LC	4	0	7	5	5	4	25 (14.8%)
	CF/FF/CC	8	3	14	13	8	19	65 (38.5%)
*An. sineroides*	LL	1	–	3	1	0	3	9 (88.9%)
	LF	0	–	0	0	1	0	1 (11.1%)

### Relative susceptibility to λ-cyhalothrin in six *Anopheles* Hyrcanus group species

The LC_50_ values of six field-collected *Anopheles* species were determined using bioassays conducted over 2 years. Subsequently, the baseline susceptibility ratio (BSR) was estimated for each species relative to the most susceptible species in each group (Table 4). *Anopheles belenrae* exhibited the highest susceptibility (LC_50_ = 36.3 ppb), followed by *An. lesteri* (LC_50_ = 218 ppb; BSR = 6.0). *Anopheles sineroides* exhibited an LC_50_ of 366 ppb (BSR = 10.1), whereas *An. kleini* and *An. pullus* showed similar levels of baseline susceptibility, with LC_50_ values of 464 ppb (BSR = 12.8) and 469 ppb (BSR = 12.9). *Anopheles sinensis* was the most resistant species, with a 75.3-fold higher LC_50_ value (2736 ppb) than *An. belenrae*. Given the averages of 91% and 84%, *An. sinensis* exhibited *kdr* mutations in the 2022 and 2023 Paju collections (Tables 2 and 3), which was likely a result of the resistance induced by the *kdr* mutation.

**Table 4:** LC_50_ and baseline susceptibility ratio (BSR) of *Anopheles* Hyrcanus group larvae at 24 h on λ-cyhalothrin treatment

spp.	Number of parental groups	Number of tested larvae	LC_50_ (ppb)	95% confidence limits	slope	*df*	*χ*^2^	BSR
*An. belenrae*	> 12	331	36.3	22.7–52.0	2.44 ± 0.34	8	27.6	1.0
*An. kleini*	4	102	464	155–1995	1.34 ± 0.51	5	10.3	12.8
*An. lesteri*	4	72	218	95.7–483	2.05 ± 0.48	8	8.23	6.0
*An. pullus*	12	184	469	250–909	1.57 ± 0.26	12	30.8	12.9
*An. sinensis*	> 12	317	2736	1424–8622	1.17 ± 0.23	8	22.3	75.3
*An. sineroides*	3	55	366	115–1609	1.67 ± 0.55	5	11.36	10.1

### Seasonal changes of susceptibility to λ-cyhalothrin in three *Anopheles* species

**Fig. 3:**
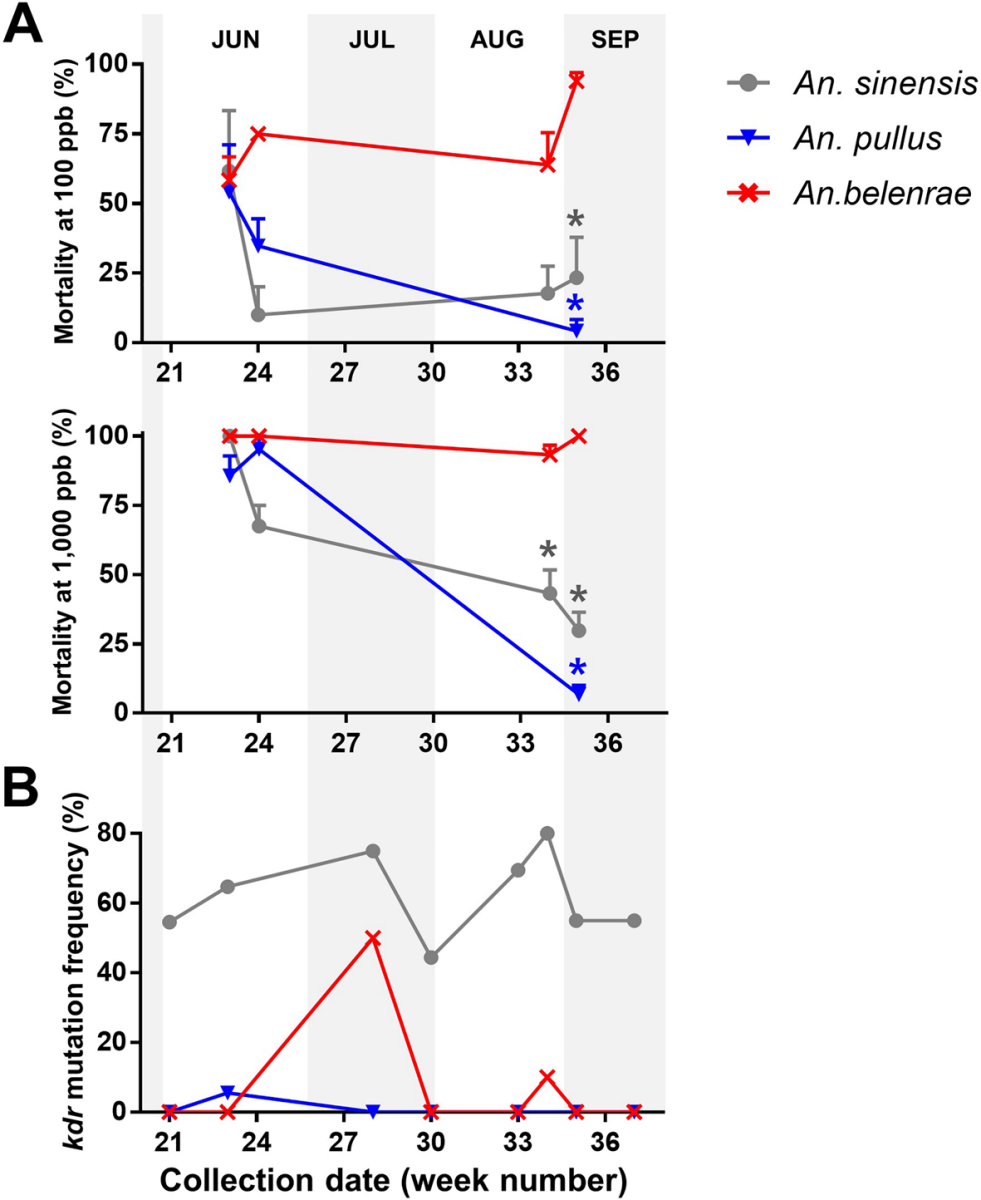
Dynamics of λ-cyhalothrin susceptibility and *kdr* mutation frequency in three *Anopheles* species in 2022, Paju. Mortality at 24 h after λ-cyhalothrin treatment at 100 and 1000 ppb (**A**) on third instar larvae was investigated. F_1_ generations of *An. belenrae*, *An. pullus*, and *An. sinensis* collected from Paju in 2022 were used for bioassays to synchronize the conditions. *X*-axis indicates the collection date of the parental generation

To track seasonal change in mosquito susceptibility, bioassays using 100 and 1000 ppb of λ-cyhalothrin (Fig. 3A) were conducted for three commonly occurring *Anopheles* species (*An. sinensis*, *An. pullus,* and *An. belenrae*) during various seasons. At the start point (week 23), the mortality of F_1_ larvae was similar across the three species, ranging from 54% to 61% at 100 ppb and 86% to 100% at 1000 ppb. Throughout the season, the mortality rate of *An. sinensis* and *An. pullus* decreased significantly, whereas *An. belenrae* maintained a constant mortality rate of > 50%. In addition, when comparing the *kdr* mutation frequency of the Paju 2022 collections with the susceptibility data (Fig. 3B), fluctuations with a double peak in mid-July and late August were observed. These fluctuations suggest the presence of selection pressure in the study area, which drives the development of pyrethroid resistance.

### Differences in VSSC protein sequences and gene copy numbers among *Anopheles* Hyrcanus group species

The full-length sequences of VSSC, obtained from 2–4 clones of each species, were submitted to GenBank (Additional file 2: Table S.2) and aligned (Additional file 3: Figs. S1–4). Although several polymorphisms were detected in some clones, no exact matches were found between any of the six *Anopheles* species and the previously reported point mutations associated with pyrethroid resistance in mosquitoes and other insects [11]. However, cross-species comparison revealed various single amino acid substitutions, the insertion of 2–40 amino acids including exon *k*, and the deletion of 8–19 amino acids including exon *h* (Table 5, [15]). Additionally, we identified nine species-specific substitutions. Among these, five were conservative replacements, suggesting that they may not affect VSSC function. The remaining four radical substitutions included A812S and K1479T, which were exclusively found in *An. pullus*, whereas H315D and N2046I were found solely in *An. lesteri*. Considering that H315D and K1479T are located in the pore regions of domains I and III, respectively, and that A812S is positioned at the beginning of S1 of domain II, they may influence the overall channel kinetics, potentially altering pyrethroid sensitivity. As duplication of the *vssc* gene can affect pyrethroid sensitivity, the copy number in individual mosquitoes was estimated (Additional file 4: Fig. S5). No significant differences were found in the average copy number among the six *Anopheles* species (analysis of variance, ANOVA, *F* = 2.258, *P* = 0.075), indicating that duplication of the *vssc* gene does not commonly occur in these populations. However, one *An. sineroides* individual showed 1.45 copies, which may suggest the presence of a heterozygous form of *vssc* duplication.

**Table 5:** Sequence variants of VSSC among *Anopheles* Hyrcanus group species

Species	Sequence variation											
	Amino acid substitution (position)									Deletion	Insertion	
	39	315	812	1076	1236	1348	1479	2042	2045	D3-4 linker	D3-s3	C-terminus
*An. belenrae*	R	H	A	M	E	L	K	T	N	–	–	–
*An. kleini*	R	H	A	M	E	L	K	T	N	–	–	–
*An. lesteri*	R	D	A	M	D*	I*	K	T	I	–	–	–
*An. pullus*	R	H	S	L*	E	I*	T	T	N	exon *h* ^1^	–	–
*An. sinensis*	R	H	A	M	E	L	K	T	N	exon *h*	exon *k* ^2^	–
*An. sineroides*	K*	H	A	M	E	L	K	S*	N	–	–	GG

^*^ This indicates the conservative substitution of amino acid

^1^^, 2^ The exon names were referred to Lee et al., 2002

### Positive association of *kdr* genotype with phenotypic λ-cyhalothrin resistance in *An. belenrae*

**Fig. 4:**
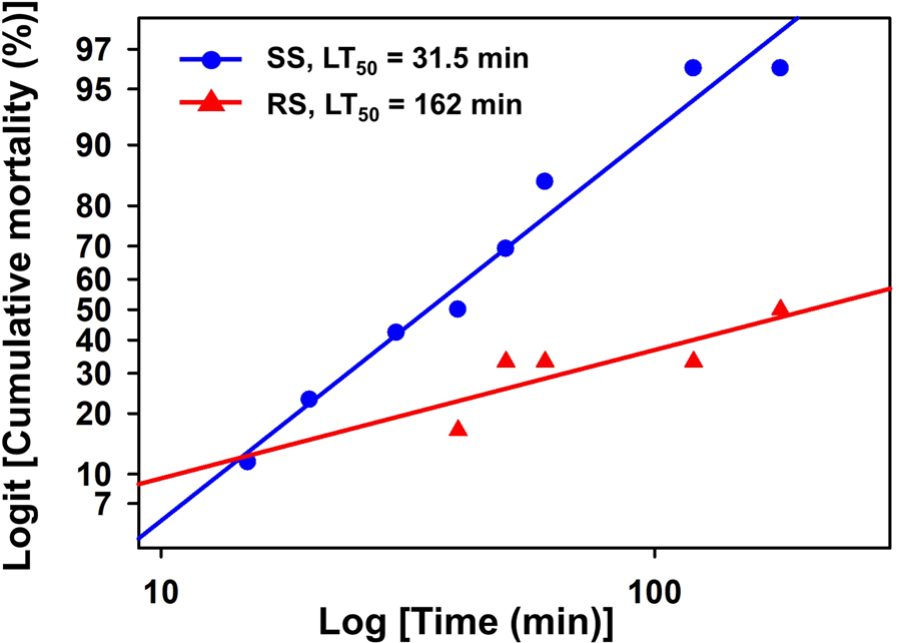
Time-mortality responses of *An. belenrae* larvae with either homozygous susceptible (SS) or heterozygous (RS) genotype. Mortality was recorded at specified time intervals following treatment with 1 ppm of λ-cyhalothrin. Scored mosquito larvae were immediately collected and their genotypes were analyzed

Excluding *An. sinensis*, *An. belenrae* showed the highest level of *kdr* mutation, reaching a maximum of 75%, although none of the individuals displayed a homozygous resistance allele. To assess the role of the *kdr* allele in pyrethroid resistance, the LT_50_ values of the two genotypes (homozygous susceptible and heterozygous) were estimated using *An. belenrae* as a model species (Fig. 4). Heterozygous individuals displayed a slower response (LT_50_ = 162 min) than susceptible homozygous individuals (LT_50_ = 31.5 min), resulting in a 5.1-fold difference. The hypothesis of equality was rejected at P < 0.001 (chi-squared test, *χ*^2^ = 36.6, *df* = 2). This result suggests that even the heterozygous allele confers phenotypic resistance and the population with a heterozygous resistance allele can survive when exposed to pyrethroid insecticides.

## Discussion

Compared with previous studies investigating mosquito species composition in the northern Gyeonggi region, significant annual variations were evident. Even after collecting data for 2 years in the same area, substantial differences persisted. Notably, there were significant changes in *An. lesteri*, from 0 individuals in 2022 to 56 individuals in 2023. *Anopheles kleini*, which is hypothesized to exhibit the highest vector competence [16], was observed only in the Paju area among the study locations, albeit at a relatively low level of up to 5.5%. Nevertheless, considering its high density of 61.8% in the 2020 survey [17], the continuous monitoring of this mosquito species is crucial.

In this study, the presence of *kdr* mutations in some *Anopheles* species, such as *An. belenrae*, *An. pullus*, *An. lesteri*, and *An. sineroides*, was newly reported through genotyping. It is important to note, however, that the small sample size in low-density species could lead to biased interpretations. For example, in week 28 of 2022 in Paju, *kdr* mutation was observed in 50% of *An. belenrae* individuals tested (Fig. 3B). Yet, when considering the entire year of 2022, only 8.7% of *An. belenrae* carried the heterozygous resistance allele. The higher percentage in week 28 was due to the fact that only one individual collected during that week carried the LF allele. The significant differences in *kdr* mutation frequency among species may be attributed to the population sizes or fitness of resistant subpopulations compared with that of susceptible subpopulations [18, 19]. Large population size and wide distribution are essential for generating a multitude of individual variations within a species. This enables sufficient genetic diversity and the spread of traits associated with insecticide resistance within the population, thereby overcoming the limitations of genetic drift [10]. Lower frequencies of *kdr* mutations in the minor *Anopheles* species than in the predominant *An. sinensis* may be attributed to their relatively low population densities, which could constrain the gene pool available for the proliferation of resistance traits. Ecological differences, such as larval habitats or adult flight ranges, may also contribute to variations in insecticide exposure. *Anopheles sinensis* mosquitoes, owing to their wide distribution, are more likely to be exposed to pyrethroids, resulting in an increased *kdr* frequency compared with other species with a more limited distribution. The absence of homozygous *kdr* allele in minor species, coupled with their relatively higher λ-cyhalothrin susceptibility, suggests that these mosquitoes are in the early stages of pyrethroid resistance development. Nevertheless, even the heterozygous genotype conferred phenotypic resistance, as determined by the LT_50_ comparison in *An. belenrae*, heterozygous individuals would have a survival advantage over susceptible homozygous individuals under pyrethroid selection pressure. Therefore, the ongoing use of pyrethroids in the field may eventually lead to the development of high levels of resistance even in minor species of *Anopheles* mosquitoes. Given that *Anopheles* mosquitoes are believed to be indirectly selected by insecticides used in agriculture or industry rather than being directly targeted by public health pyrethroids [20], continuous monitoring of agricultural and industrial pyrethroids, along with effective management of mosquito habitats, is crucial for mitigating selection pressure against *Anopheles* mosquitoes. Because *kdr* mutations are known to incur high fitness costs in many insects [21, 22], including outdoor mosquitoes [13], understanding the fitness cost associated with *kdr* mutations in individual species of the *Anopheles* Hyrcanus group is imperative for efficient resistance management.

This study represents the first comparison of baseline susceptibility to pyrethroid insecticides among coexisting species. Despite continuous exposure to pyrethroid insecticides, significant differences in baseline susceptibility were observed among species. *Anopheles sinensis* exhibited a 75-fold reduction in λ-cyhalothrin susceptibility compared with *An. belenrae*. This remarkably low baseline susceptibility of *An. sinensis* appears to be primarily attributable to its high *kdr* mutation frequency (62%). Additionally, the presence of a transcription variant with a 41-bp insertion in transmembrane segment 3 of *An. sinensis* VSSC homology domain III, known as exon *k* [23], likely contributed to the further reduction in pyrethroid sensitivity, as supported by functional expression studies [24].

*Anopheles pullus* and *An. kleini* exhibited an approximately 13-fold reduction in susceptibility compared with *An. belenrae*; however, the *kdr* mutation frequencies were significantly lower than those of *An. sinensis*. Species-specific substitutions in the *An. pullus* VSSC may reduce its λ-cyhalothrin susceptibility. Nevertheless, the VSSC amino acid sequence of *An. kleini* was not significantly different from that of *An. belenrae*, and the observed difference in λ-cyhalothrin susceptibility between the two species was not due to any differential target site sensitivity. Taken together, these findings suggest that other resistance mechanisms, such as metabolic or cuticular penetration factors, may be involved in the cross-species differences in susceptibility, rather than the target site insensitivity mechanism, including the *kdr* mutation. Therefore, investigating the intrinsic differences in the genetic and physiological properties that govern insecticide susceptibility and their correlation with vector competence among species is essential to gaining a comprehensive understanding of the species-specific susceptibility status.

Due to the difficulty in obtaining synchronized adult specimens, baseline susceptibility to pyrethroids in this study was assessed by measuring larval susceptibility to λ-cyhalothrin. However, because pyrethroid insecticides are not typically used as larvicides, larval assays may not perfectly reflect the pyrethroid susceptibility levels of adult mosquitoes.

Insecticide resistance in vector mosquitoes significantly affects the transmission of vector-borne diseases by influencing their longevity and reproductive capacity. Numerous studies have demonstrated the correlation between the prevalence and transmission rates of vector-borne diseases and insecticide resistance in *An. gambiae*, a tropical malaria vector [25]. Interestingly, among domestic vector species, *An. kleini,* which exhibits relatively high vector competence [16], showed considerable resistance to pyrethroids despite the absence of *kdr* mutation. This finding prompted speculation that the potentially enhanced metabolic detoxification by *An. kleini* may be associated with increased vector competence. This association suggests a trade-off between its capacity for insecticide detoxification and the immune response, potentially leading to reduced immunity against parasitic infection. Further comprehensive research is required to understand the relationship between vector competence and insecticide resistance.

## Conclusions

We investigated the distribution of the *Anopheles* Hyrcanus group, a vector of vivax malaria in South Korea, along with their pyrethroid susceptibility status. *Anopheles sinensis*, the most prevalent species, developed resistance most likely through target-site mutations. Our research also revealed diverse *kdr* mutation frequencies and notable variations in susceptibility to pyrethroid insecticides among previously overlooked minor species. Specifically, low frequencies of *kdr* mutations were identified in *An. belenrae*, *An. lesteri*, *An. pullus*, and *An. sineroides* for the first time, providing evidence for the development of initial resistance. Given that pyrethroid insecticides remain the primary choice for mosquito control, further studies on the resistance mechanisms, particularly those focusing on metabolic resistance, are crucial for these species. Furthermore, the effective management of mosquito vector populations is contingent on various factors, including seasonal density and resistance to insecticides. Thus, enhancing surveillance systems on the basis of species-specific phenology and seasonal dynamics of resistance is crucial for better responsiveness to ongoing climate change and effectively curtailing malaria transmission.

## Supplementary Information

Additional file 1: Tables S.1 Multiplex PCR primer sets used for species identification of six *Anopheles *Hyrcanus group species.

Additional file 2: Tables S.2 GenBank accession numbers of cloned voltage-sensitive sodium channel sequences of six *Anopheles* Hyrcanus group.

Additional file 3: Figures S.1–4 The deduced protein sequence alignment of the *vssc* gene encompassing domains I–IV of the six species belonging to the *Anopheles* Hyrcanus group with *An. sinensis* obtained from VectorBase. The start and end points of each domain are indicated by red arrows and the blue boxes indicate segments 1–6 within each domain. Sequence variations are denoted by a yellow background, with species-specific variations highlighted by red boxes.

Additional file 4: Figure S.5 The copy number of *vssc* gene estimated using the single copy genes (*RPS3* and *RPS7*) in six *Anopheles* Hyrcanus group species.

## Data Availability

The nucleotide and amino acid sequences generated in this study have been deposited in the GenBank database under the accession numbers (PQ136754-PQ136840). These sequences include the partial voltage-sensitive sodium channel (*vssc*) genes from six *Anopheles* Hyrcanus Group species.

## Abbreviations

*kdr*: Knockdown resistance
VSSC: Voltage-sensitive sodium channel
pPCR: Quantitative real-time polymerase chain reaction
LC_50_: Median lethal concentration
LT_50_: Median lethal time
BSR: Baseline susceptibility ratio
