# Supplementary material for: Differences in seasonal dynamics and pyrethroid resistance development among Anopheles Hyrcanus group species
Source: Parasit Vectors. 2024 Oct 5;17:417. doi: 10.1186/s13071-024-06462-8 (PMC11456232; doi:10.1186/s13071-024-06462-8)
Supplement: Supplementary file 1 — Additional file 1: Tables S.1 Multiplex PCR primer sets used for species identification of six Anopheles Hyrcanus group species. [file 13071_2024_6462_MOESM1_ESM.docx]

**Table S.1** Multiplex PCR primer sets used for species identification of six *Anopheles* Hyrcanus Group species

| Primer name | Target species | Sequence (5’-3’) | Expected size (bp) | Reference |
| --- | --- | --- | --- | --- |
| An_5.8S-F | Universal | TGTGAACTGCAGGACACATGAA |  | Li et al., 2005 |
| Bel_ITS2-R | *An. belenrae* | CATTTTTCACGACTGCGACGG | 162 | Li et al., 2005 |
| Pul_ITS2-R | *An. pullus* | TTG ATA TCA TGG CTT AAC ACC G | 227 | Li et al., 2005 |
| Les_ITS2-R | *An. lesteri* | GC CCA TTC CAC TAT CTC GAA G | 390 | This study |
| Kle_ITS2-R | *An. kleini* | CTTGTATCGTCCATCCGCTATA | 481 | This study |
| Sin_ITS2-R | *An. sinensis* | AGTGGCCTCACTCTTGGAG | 541 | This study |
| Srd_28S-R | *An. sineroides* | AAATCCAGGTTGCGGGCATC | 1,005 | This study |
