# Supplementary material for: Differences in seasonal dynamics and pyrethroid resistance development among Anopheles Hyrcanus group species
Source: Parasit Vectors. 2024 Oct 5;17:417. doi: 10.1186/s13071-024-06462-8 (PMC11456232; doi:10.1186/s13071-024-06462-8)
Supplement: Supplementary file 2 — Additional file 2: Tables S.2 GenBank accession numbers of cloned voltage-sensitive sodium channel sequences of six Anopheles Hyrcanus group. [file 13071_2024_6462_MOESM2_ESM.docx]

**Table S.2** GenBank accession numbers of cloned voltage-sensitive sodium channel sequences of six *Anopheles* Hyrcanus Group

| Sequence region | Species | Accession number | Sequence region | Species | Accession number |
| --- | --- | --- | --- | --- | --- |
| Domain I | *An. belenrae* | PQ136754-PQ136755 | Domain III | *An. belenrae* | PQ136773- PQ136775 |
|  | *An. kleini* | PQ136756-PQ136759 |  | *An. kleini* | PQ136776-PQ136778 |
|  | *An. lesteri* | PQ136760- PQ136761 |  | *An. lesteri* | PQ136779-PQ136780 |
|  | *An. pullus* | PQ136762- PQ136765 |  | *An. pullus* | PQ136781-PQ136783 |
|  | *An. sinensis* | PQ136766- PQ136768 |  | *An. sinensis* | PQ136784-PQ136786 |
|  | *An. sineroides* | PQ136769-PQ136772 |  | *An. sineroides* | PQ136787-PQ136789 |
| Domain II | *An. belenrae* | PQ136790-PQ136792 | Domain IV | *An. belenrae* | PQ136810-PQ136811 |
|  | *An. kleini* | PQ136793-PQ136796 |  | *An. kleini* | PQ136812-PQ136815 |
|  | *An. lesteri* | PQ136797-PQ136798 |  | *An. lesteri* | PQ136816-PQ136818 |
|  | *An. pullus* | PQ136799-PQ136802 |  | *An. pullus* | PQ136819-PQ136822 |
|  | *An. sinensis* | PQ136803-PQ136805 |  | *An. sinensis* | PQ136823-PQ136826 |
|  | *An. sineroides* | PQ136806-PQ136809 |  | *An. sineroides* | PQ136827-PQ136830 |
