# Supplementary figures and images for: Differences in seasonal dynamics and pyrethroid resistance development among Anopheles Hyrcanus group species
Source: Parasit Vectors. 2024 Oct 5;17:417. doi: 10.1186/s13071-024-06462-8 (PMC11456232; doi:10.1186/s13071-024-06462-8)

Fig 1


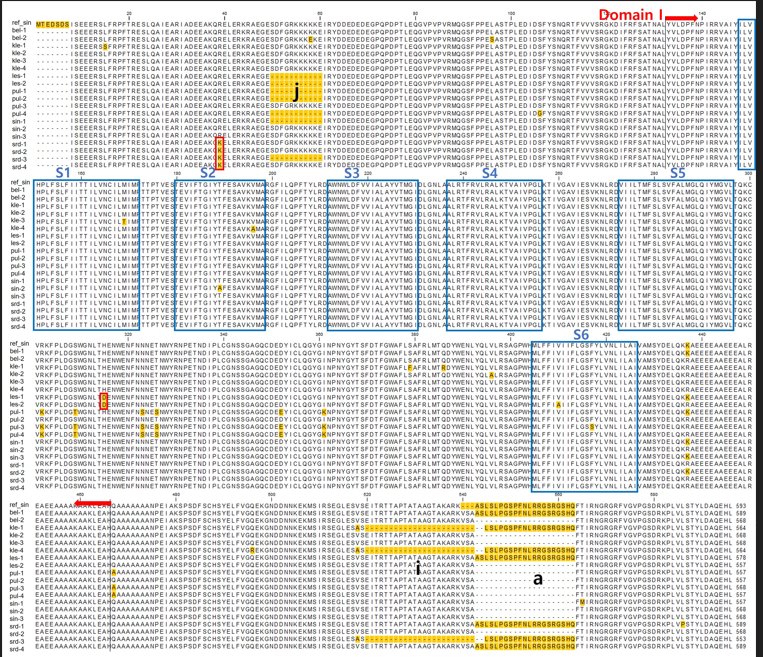


Fig 2


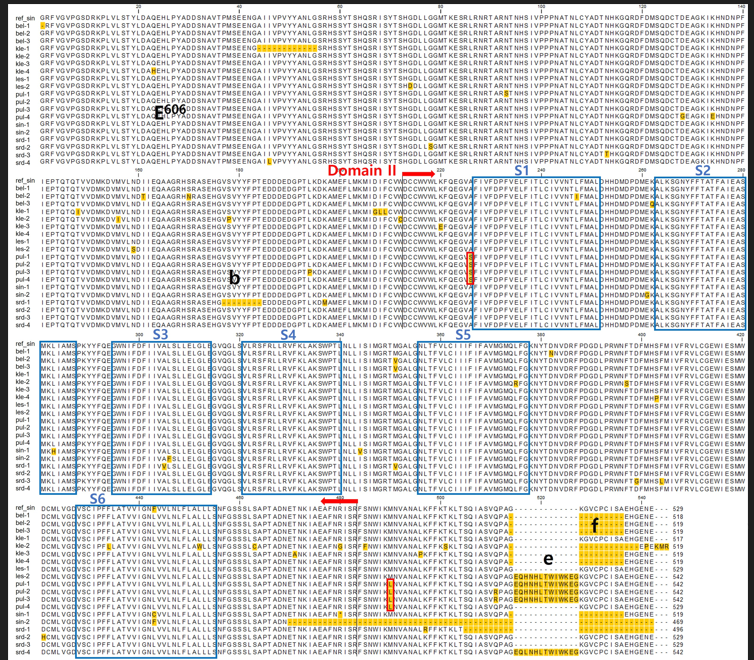


Fig 3


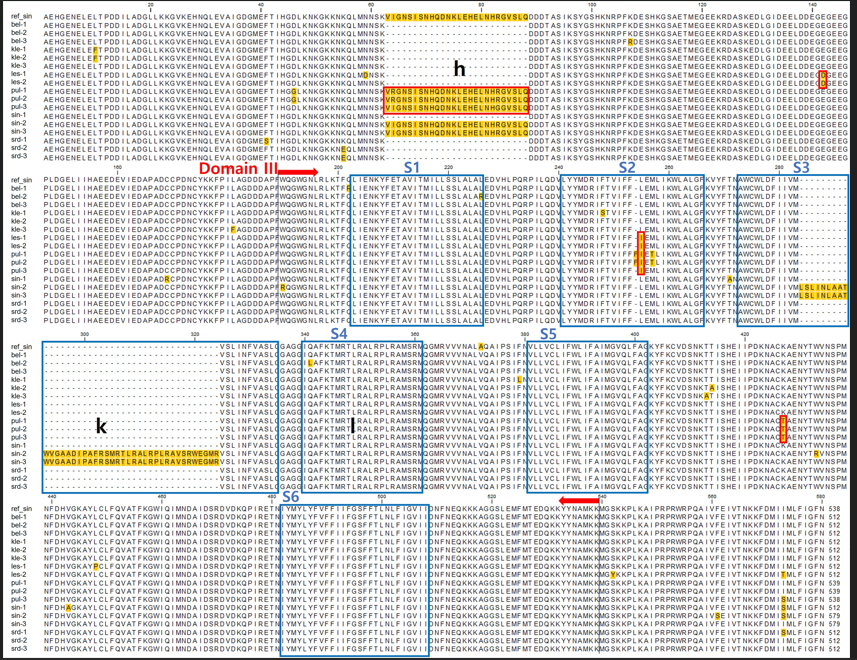


Fig 4


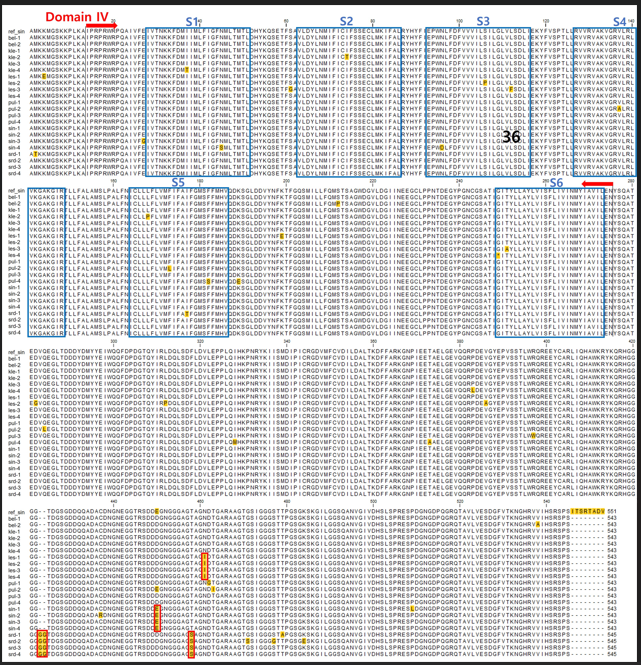

Supplement: Supplementary file 3 — Additional file 3: Figures S.1–4 The deduced protein sequence alignment of the vssc gene encompassing domains I–IV of the six species belonging to the Anopheles Hyrcanus group with An. sinensis obtained from VectorBase. The start and end points of each domain are indicated by red arrows and the blue boxes indicate segments 1–6 within each domain. Sequence variations are denoted by a yellow background, with species-specific variations highlighted by red boxes. [file 13071_2024_6462_MOESM3_ESM.docx]

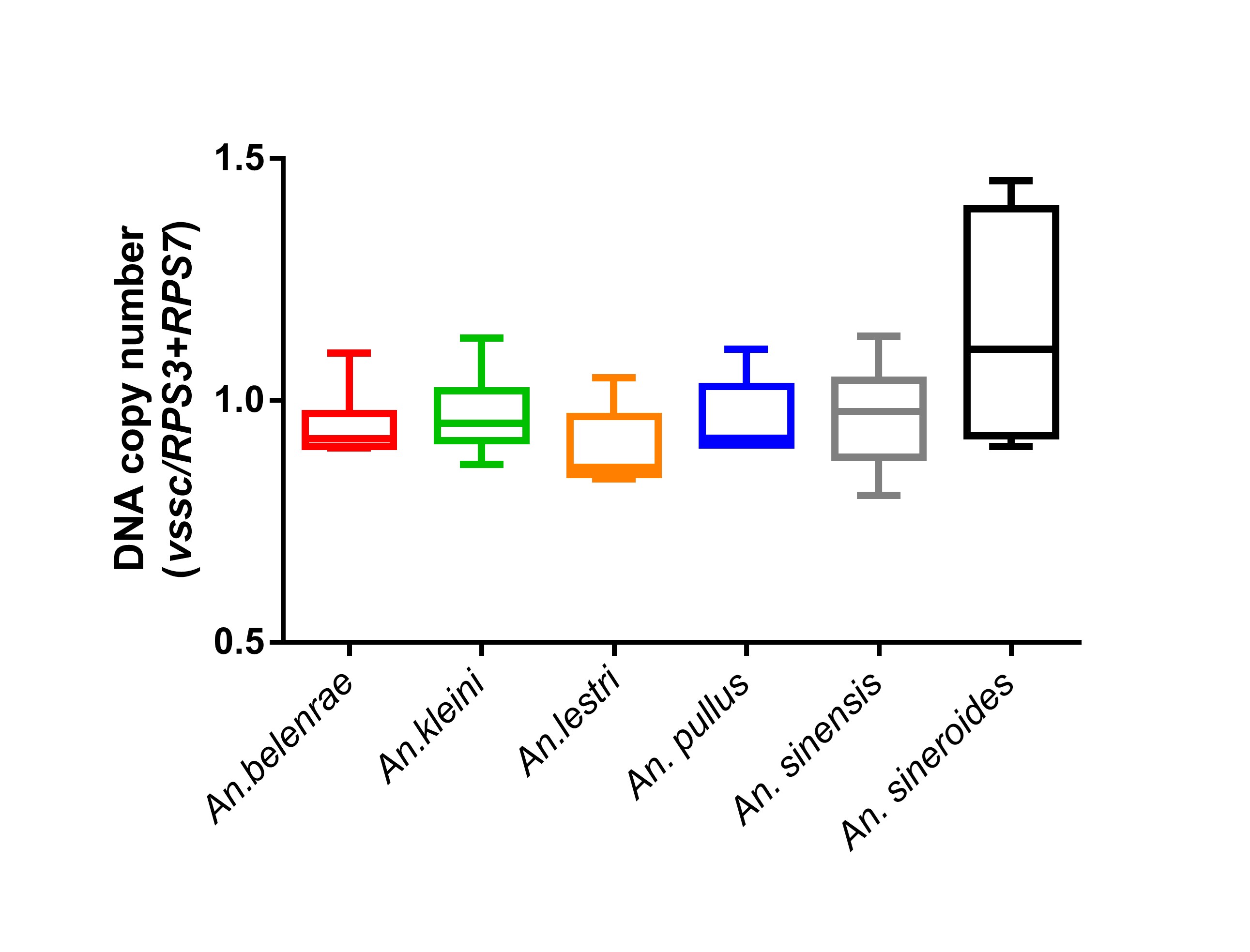

Supplement: Supplementary file 4 — Additional file 4: Figure S.5 The copy number of vssc gene estimated using the single copy genes (RPS3 and RPS7) in six Anopheles Hyrcanus group species. [file 13071_2024_6462_MOESM4_ESM.jpg]
